# Ultrastructural characterization of microlipophagy induced by the interaction of vacuoles and lipid bodies around generative and sperm cells in *Arabidopsis* pollen

**DOI:** 10.1007/s00709-020-01557-2

**Published:** 2020-09-23

**Authors:** Kae Akita, Tomoko Takagi, Keiko Kobayashi, Kazuyuki Kuchitsu, Tsuneyoshi Kuroiwa, Noriko Nagata

**Affiliations:** 1 Department of Chemical Biological Sciences, Faculty of Science, Japan Women’s University, Bunkyo-ku, Tokyo, Japan; 2 Department of Applied Biological Science, Tokyo University of Science, Noda, Chiba Japan

**Keywords:** *ATG2*, Lipid body, Lipophagy, Microautophagy, Pollen, Vacuole

## Abstract

During pollen maturation, various organelles change their distribution and function during development as male gametophytes. We analyzed the behavior of lipid bodies and vacuoles involved in lipophagy in *Arabidopsis* pollen using serial section SEM and conventional TEM. At the bicellular pollen stage, lipid bodies in the vegetative cells lined up at the surface of the generative cell. Vacuoles then tightly attached, drew in, and degraded the lipid bodies and eventually occupied the space of the lipid bodies. Degradation of lipid began before transfer of the entire contents of the lipid body. At the tricellular stage, vacuoles instead of lipid bodies surrounded the sperm cells. The degradation of lipid bodies is morphologically considered microautophagy. The *atg2-1 Arabidopsis* mutant is deficient in one autophagy-related gene (*ATG*). In this mutant, the assembly of vacuoles around sperm cells was sparser than that in wild-type pollen. The deficiency of ATG2 likely prevents or slows lipid degradation, although it does not prevent contact between organelles. These results demonstrate the involvement of microlipophagy in the pollen development of *Arabidopsis*.

**Electronic supplementary material:**

The online version of this article (10.1007/s00709-020-01557-2) contains supplementary material, which is available to authorized users.

## Introduction

Several ultrastructural studies show that various organelles dramatically change shape, structure, and distribution during pollen development (Marciniec et al. 2019; Nagata 2010; Paul et al. 2016; Tchórzewska 2017). For example, pollen grains contain large numbers of lipid storage bodies. Lipid bodies are organelles enclosed by a single membrane that mainly contain lipid esters, i.e., triacylglycerols and cholesteryl esters. These bodies participate in the modulation of neutral lipid metabolism (Fujimoto and Parton 2011). Lipidic organelles are generally identified as lipid droplets. Although known by a variety of names, we will refer to them as lipid bodies in this study.

After the first pollen mitosis in *Arabidopsis*, numbers of lipid bodies begin to increase, which coincides with rapid production of cytoplasm to fill most of the vegetative cell (Owen and Makaroff 1995). The lipid bodies appear to play a key role in development as male gametophytes. The amount of triacylglycerol in pollen increases until the second pollen mitosis but decreases considerably before flowering (Piffanelli et al. 1998). Vegetative cells in pollen grains must deliver sperm cells to the ovule by producing a pollen tube. Tube formation may require a supply of fatty acid and membrane lipid from lipid bodies. Yamamoto et al. (2003) reported the following unique changes of vacuoles during *Arabidopsis* pollen development: a large vacuole was divided into small vacuoles after the first mitosis; somatic-type vacuoles disappeared after the second mitosis; and membrane-bound structures containing fine fibrillar substances (MBFs) appeared in mature pollen grains and then changed to lytic vacuoles. Thus, vacuoles also change dramatically during pollen development and are expected to be involved in the degradation of lipid bodies and supply of lipid components.

Evidence from both yeast and mammalian cells shows that lipid droplets may interact with other structures, and lipid exchange often occurs between lipid droplets and various organelles to regulate lipid homeostasis (Gao and Goodman 2015). The role of autophagy in degradation of various organelles has been known for some time, but the contribution of autophagy to lipid droplet degradation has only been recently identified. “Lipophagy” is defined as autophagic degradation of lipid droplets. The process can occur via both macro- and micromechanisms (Schulze et al. 2017; Tarique et al. 2019). Macroautophagy refers to the process where a double membrane vesicle (autophagosome) is formed to enclose a portion of the cytoplasm. Subsequently, the autophagosome fuses with vacuole/lysosomal membrane with its outer membrane and releases its cargo surrounded by the inner membrane. Microautophagy, in contrast, is the direct and transient interaction of vacuoles/lysosomes with cellular compartments, such as organelles. Some types of microautophagy may recognize membrane deformation, but microautophagy is considered to be a type of lysosomal invagination (Oku and Sakai 2018). The regulation of lipophagy in higher plants is not well understood (Huang et al. 2019); a recent report indicated that lipophagy occurs in a process that morphologically resembles macrolipophagy and requires core components of macroautophagy machinery (Fan et al. 2019).

Macroautophagy is tightly regulated by a conserved set of proteins coded in autophagy-related genes (ATGs) in various eukaryotes. In contrast, microautophagy may involve both ATG-dependent and ATG-independent pathways. For example, micropexophagy (microautophagy targeting peroxisome) and microlipophagy are reported to require a series of ATG proteins in yeast (Sieńko et al. 2020). ATG-dependent microautophagy in plants was reported, in which chloroplasts damaged by high light are selectively eliminated (Nakamura and Izumi 2019). However, ATG activity is not essential for microautophagic degradation of yeast lipid droplets induced by inhibition of phosphatidylcholine biosynthesis (Vevea et al. 2015).

Plant ATG proteins are organized like yeast and mammalian ATG pathways. *ATG2*, a representative gene within *ATGs*, is involved in the early steps of autophagosome biogenesis in macroautophagy. An *atg2 Arabidopsis* mutant exhibits an over-accumulation of autophagic vesicles under nitrogen starvation (Kang et al. 2018). Thus, plant autophagy is well known to occur under stress, such as nutrient-deficient conditions (Aubert et al. 1996; Bassham et al. 2006). In contrast, even if *atg2 Arabidopsis* mutants are grown in nutrient-rich conditions, early senescence and excessive immunity-related programmed cell death occur. These effects may be related to salicylic acid signaling (Yoshimoto et al. 2009). Unlike mammals, *Arabidopsis* autophagy–deficient mutants are capable of producing offspring, and their life cycle appears normal, although high-temperature stress impairs pollen development in these mutants (Dündar et al. 2019). In contrast, rice mutants defective in autophagy show sporophilic male sterility and immature pollen, indicating crucial roles for autophagy during pollen maturation (Hanamata et al. 2014; Kurusu et al. 2014; Kurusu and Kuchitsu 2017).

Recent advances for observation of ultrathin sections using scanning electron microscopy (SEM) make the technique more suitable for observing a wider area without damage. This process allows 3D structures to be identified more easily than when using transmission electron microscopy (TEM). This new method is called serial section SEM or array tomography (Koga et al. 2016; Micheva and Smith 2007). *A. thaliana* is the most used species for the study of plant biology and understanding the details of pollen structure. Thus, we re-examined the ultrastructure of *Arabidopsis* pollen using new SEM methods. We focused our attention on lipid bodies and vacuoles and report ultrastructural changes during pollen development, resulting in new information on lipophagy within pollen.

## Material and methods

### Plant materials

*Arabidopsis thaliana* ecotype Columbia was used in this study. The *atg2-1* (SALK_076727) mutant was obtained from the ABRC. Seeds were sown on one-half MS medium (Wako Pure Chemical Industries, Ltd., Japan) supplemented with 1.5% (w/v) sucrose and stored at 4 °C for more than 2 days. After vernalization, plants were grown for 2 weeks on one-half MS agar medium and were then transferred to soil. Plants were cultured for 40–50 days. All growth occurred under a 16:8-h light/dark cycle at 23 °C in a growth chamber.

### Electron microscopy

*Arabidopsis* anthers were fixed in 4% glutaraldehyde and 4% paraformaldehyde buffered with 50 mM sodium cacodylate at pH 7.0 overnight at 4 °C and washed with the same buffer for 4 h at 4 °C. Subsequently, anthers were post-fixed with 2% OsO_4_ in 50 mM cacodylate buffer for 2 h at 4 °C. Fixed samples were dehydrated in an alcohol series and embedded in Spurr’s resin (Polysciences Inc., PA, USA). Ultrathin sections (80 nm) were cut with a diamond knife (Diatome, Biel, Switzerland) on an ultramicrotome (Ultracut S; Leica, Vienna, Austria).

For TEM analysis, sections were transferred to Formvar-coated grids and double-stained with 4% uranyl acetate for 12 min and with lead citrate solution for 3 min. After washing with distilled water, the samples were visualized using a transmission electron microscope (JEM-1200 or JEM-1400; Jeol, Tokyo, Japan) with an accelerating voltage of 80 or 100 kV.

For serial section SEM analysis, the floating serial sections were picked up and mounted on a cover glass (13 mm circle; Matsunami Glass Ind., Ltd., Osaka, Japan). Sections were attached to cover glasses by drying. The sections were double-stained with 0.4% uranyl acetate for 10 min and lead citrate solution for 3 min. Subsequently, the cover glass was coated with an osmium coater (Neoc Pro; Meiwafosis Co., Ltd., Tokyo, Japan). Serial sections were observed on an SEM with a highly sensitive BSE detector (SU8220; Hitachi, Tokyo, Japan) and an accelerating voltage of 2 kV.

### Three-dimensional reconstruction of serial ultrathin section images

Serial ultrathin section images were manually aligned using Adobe Photoshop software (Photoshop CC; Adobe Systems Inc., CA, USA). Lipid bodies and vacuoles were manually segmented by tracing the boundary contours, and gaps between images were properly filled. Images were then imported into ImageJ software, and 3D reconstructed volume rendering images were created (Schneider et al. 2012).

### Data analysis

Regions of vacuoles, lipid bodies, generative cells, and sperm cells were segmented with manual modifications to the results of ImageJ Fiji plug-in, Trainable Weka Segmentation (Arganda-Carreras et al. 2017; Schindelin et al. 2012). Distances from generative or sperm cell to each vacuole or lipid body in segmented regions were obtained using the ImageJ Distance Map (Schneider et al. 2012).

## Results

### Characteristic distribution of vacuoles and lipid bodies in pollen

**Fig. 1:**
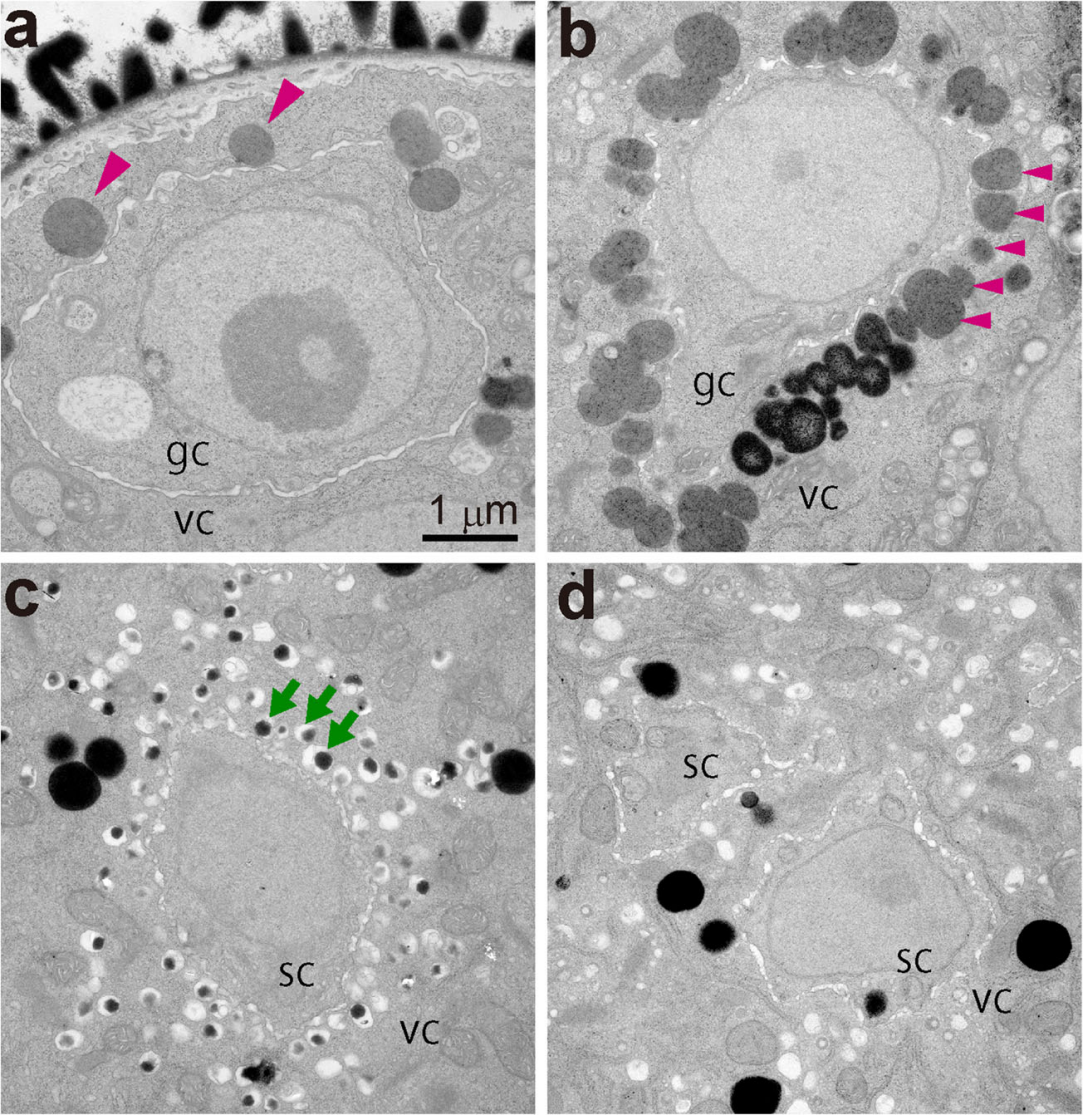
Ultrastructural analysis of the distribution of vacuoles and lipid bodies in pollen grains during pollen development by TEM. **a** Early bicellular pollen stage before generative cells move away from the pollen wall. **b** Middle bicellular pollen stage, showing accumulation of lipid bodies in the vegetative cell cytoplasm at the surface of the generative cell. **c** Relatively early tricellular pollen stage, showing accumulation of vacuoles at the surface of the sperm cell. **d** Mature tricellular pollen stage. Green arrows indicate vacuoles around the sperm cell, and magenta arrowheads indicate lipid bodies around the generative cells. gc, generative cells; sc, sperm cells; vc, vegetative cells. **a**–**d** have the same magnification

A remarkable feature of organelles in *Arabidopsis* pollen is the abundance of lipid bodies. We observed ultrastructure during pollen development using TEM (Fig. 1). In the early stages of bicellular pollen before generative cells migrate away from the pollen wall, lipid body localization is sparse and not characteristic (Fig. 1a). At the subsequent bicellular pollen stage, lipid bodies show characteristic accumulation in vegetative cell cytoplasm at the surface of the generative cell (Fig. 1b). The alignment of lipid bodies at the surface on the generative cells is transient during the bicellular pollen stage. At a relatively early tricellular stage, vacuoles instead of lipid bodies appear to surround sperm cells (Fig. 1c). In mature pollen, that is, late tricellular stage, neither vacuoles nor lipid bodies are particularly prevalent around sperm cells (Fig. 1d).

**Fig. 2:**
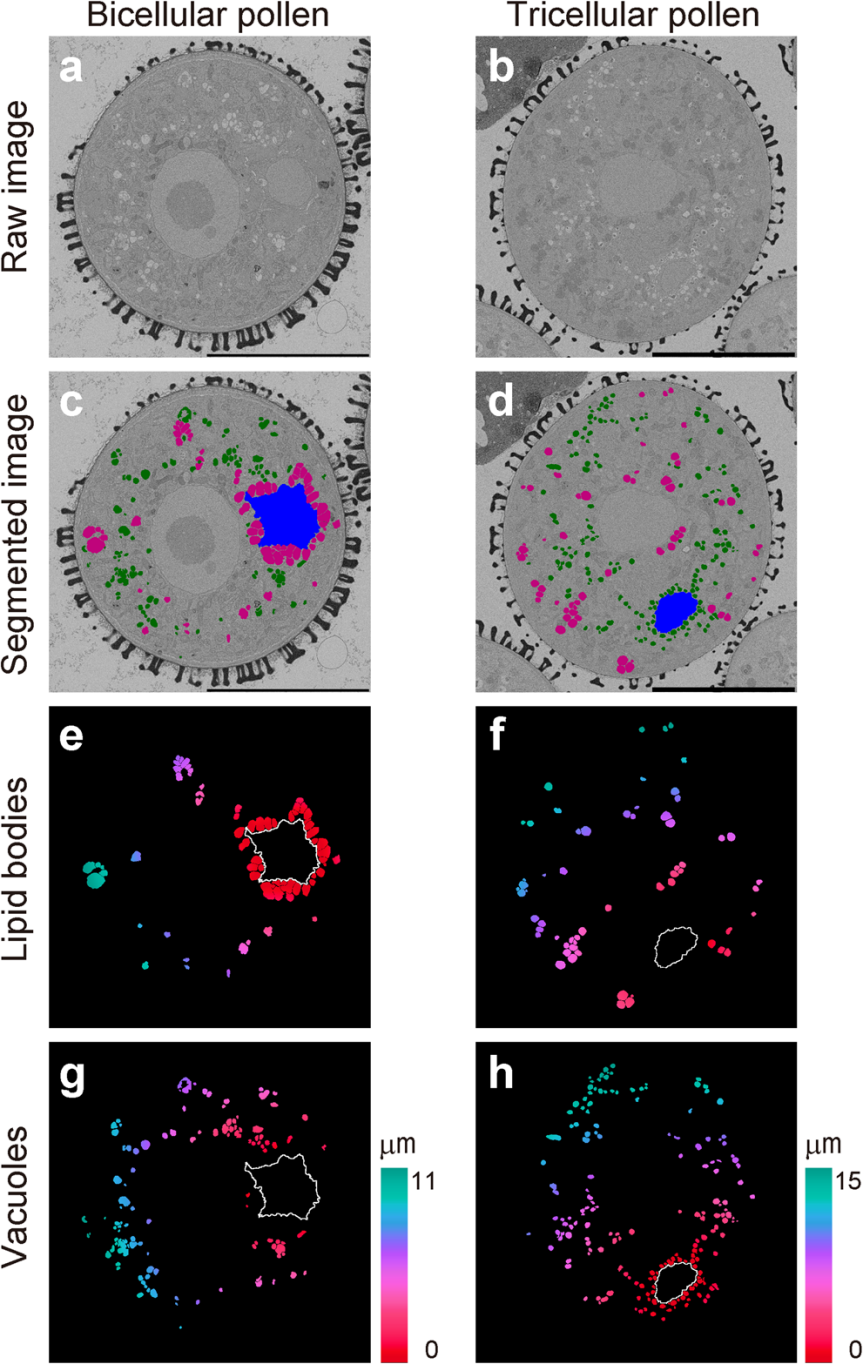
Distance measurements from generative or sperm cells to each organelle. **a**, **b** SEM images used to analyze localization. **c**, **d** Segmented regions of vacuoles (green), lipid bodies (magenta), and generative or sperm cell (blue). **e**–**h** Results of distance transformation as a color gradient from generative cell to lipid bodies (**e**) and vacuoles (**g**) or from sperm cell to lipid bodies (**f**) and vacuoles (**h**). The images corresponding to the bicellular stage in Fig. 1b (**a**, **c**, **e**, **g**) and the tricellular stage in Fig. 1c**(b**, **d**, **f**, **h**) are shown. White lines indicate the outline of the generative (**e**, **g**) or sperm cell (**f**, **h**). Bar = 10 μm

We used SEM to investigate the ultrastructure of large areas to visualize organelle distribution (Fig. 2). Pollen stage in Fig. 2a corresponds to bicellular stage in Fig. 1b, and that in Fig. 2b corresponds to the tricellular stage in Fig. 1c. Quantitative understanding of the spatial distribution of vacuoles and lipid bodies in pollen grains was assessed by assigning regions of vacuoles to green lipid bodies to magenta and generative or sperm cells to blue (Fig. 2c, d). We also visualized the distance from generative cell to vacuoles or lipid bodies, from sperm cells to lipid bodies or vacuoles as color gradients (Fig. 2e–h). Apparently, more lipid bodies are present at the bicellular stage and more vacuoles around the generative/sperm cells at the tricellular stage, although both organelles are interspersed in the vegetative cytoplasm within pollen at both stages. Sizes of vacuoles and lipid bodies are reduced between bicellular and tricellular stage.

**Fig. 3:**
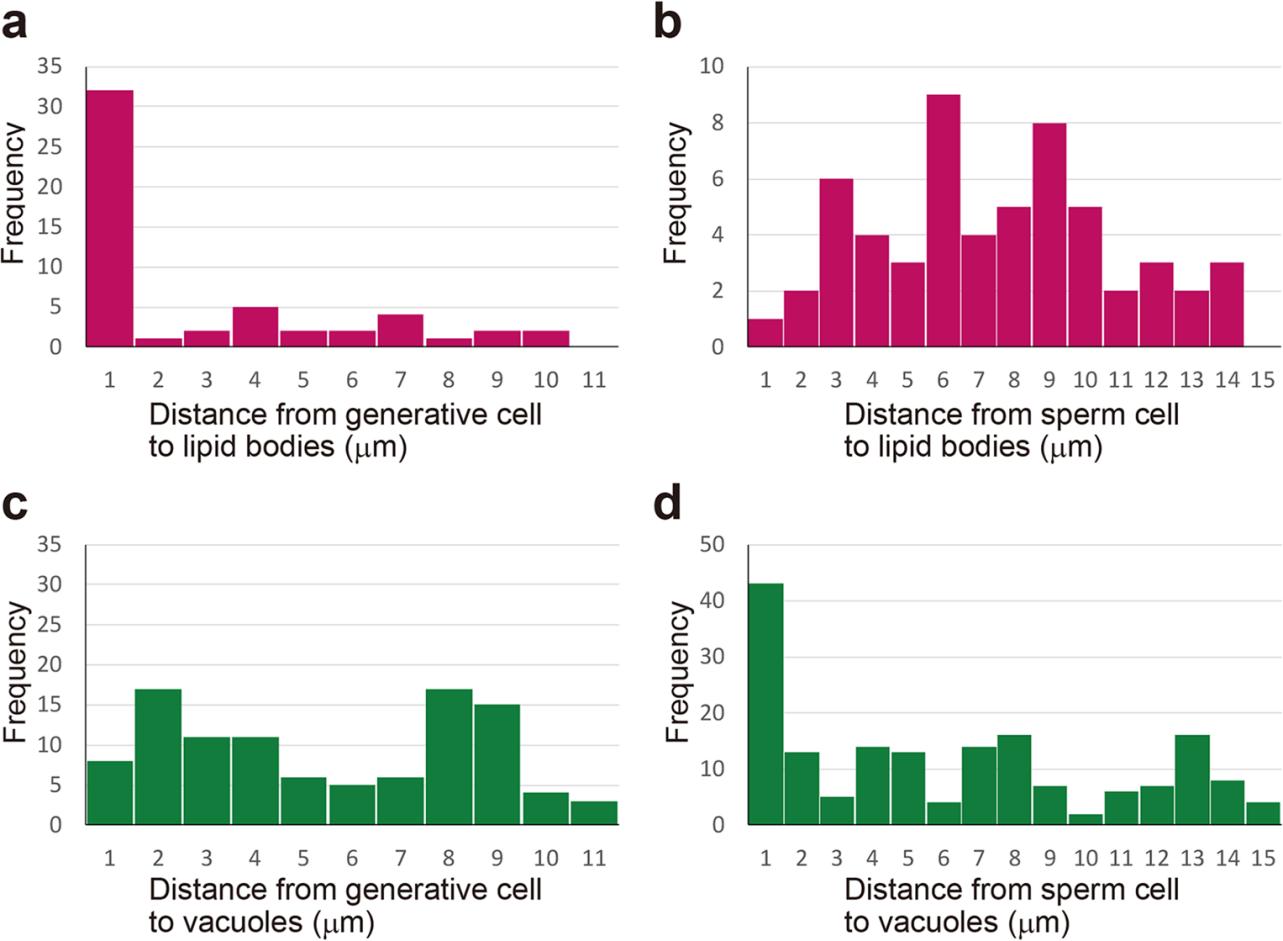
Histogram of distances of organelles from generative or sperm cell. Lipid bodies; *n* = 53 in bicellular pollen (**a**), *n* = 57 in tricellular pollen (**b**). Vacuole; *n* = 103 in bicellular pollen (**c**), *n* = 172 in tricellular pollen (**d**). Note that in the bicellular pollen, 60% of lipid bodies were localized around the generative cell, whereas in tricellular pollen, they were distributed throughout the cell. Conversely, 25% of vacuoles accumulated around sperm cells in tricellular pollen

Figure 3 shows histogram the distance of organelle from generative or sperm cells. In bicellular pollen, 60% of lipid bodies were localized around the generative cell, whereas in tricellular pollen, they were distributed throughout the cell. In contrast, 25% of vacuoles accumulated around the sperm cell in tricellular pollen. The distribution of these organelles differs. Numbers of vacuoles were 103 and 172, and that of lipid bodies were 53 and 57 for bicellular and tricellular stages, respectively. The number of vacuoles was significantly increased. Lipid bodies around the generative cell appear to have been replaced by vacuoles, which then appear to increase in number.

### Microlipophagy caused by direct interaction between lipid bodies and vacuoles

**Fig. 4:**
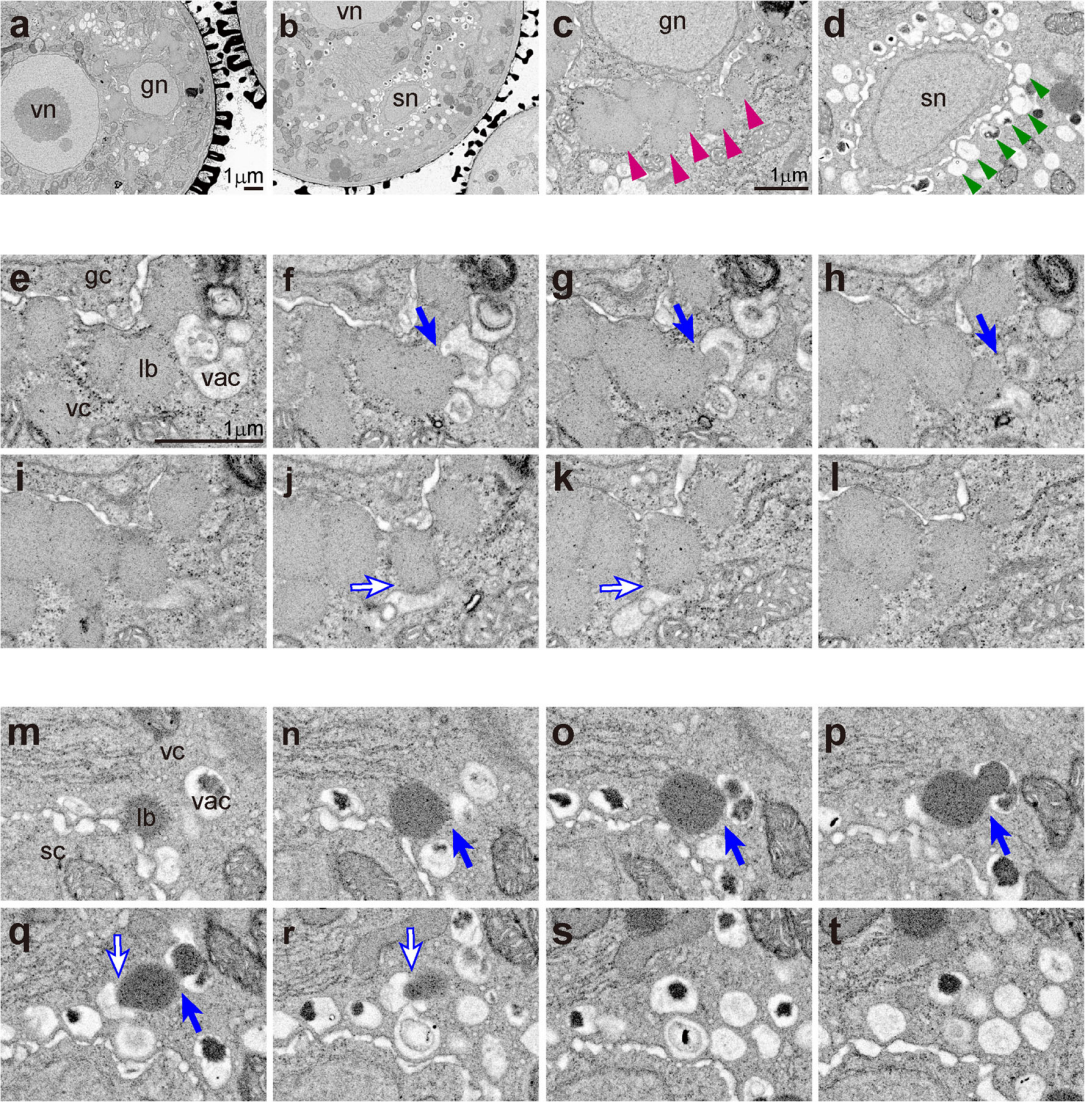
Ultrastructural analysis of interactions of vacuoles and lipid bodies by serial section SEM. **a**–**d** Enlarged images at bicellular pollen (**a**, **c**) of Fig. 2a and tricellular pollen (**b**, **d**) of Fig. 2b. **e**–**t** Serial section SEM images of the bicellular stage (**e**–**l**) and the tricellular stage (**m**–**t**). Magenta arrowheads indicate lipid bodies aligned around the generative cell, and green arrowheads indicate vacuoles aligned around the sperm cell. Arrows indicate interaction regions between vacuoles and lipid bodies. Note that vacuoles appear to draw in lipid bodies. gc, generative cells; gn, generative nuclei; lb, lipid bodies; sc, sperm cells; sn, sperm nuclei; vac, vacuoles; vc, vegetative cells; vn, vegetative nuclei. **a**–**b**, **c**–**d**, and **e**–**t** have the same magnification, respectively

Vacuoles may be involved in the degradation of lipid bodies. We observed serial sections using SEM (Fig. 4) to examine correlations between organelles. SEM images of serial sections (Fig. 4e–l) of pollen grains at the bicellular stage (Fig. 2a) captured a vacuole directly attached to a large lipid body. Interactions between vacuole and lipid body are indicated with arrows in Fig. 4. Vacuoles appear to adhere tightly to lipid bodies, with invagination of vacuolar membranes (tonoplast). The tricellular stage (Fig. 2b) showed this same phenomenon (Fig. 4m–t). Vacuoles attached tightly and drew lipid directly in two locations on a large lipid body. Vacuoles in tricellular pollen often contained electron-dense areas that appear to be residues of degraded lipid bodies.

**Fig. 5:**
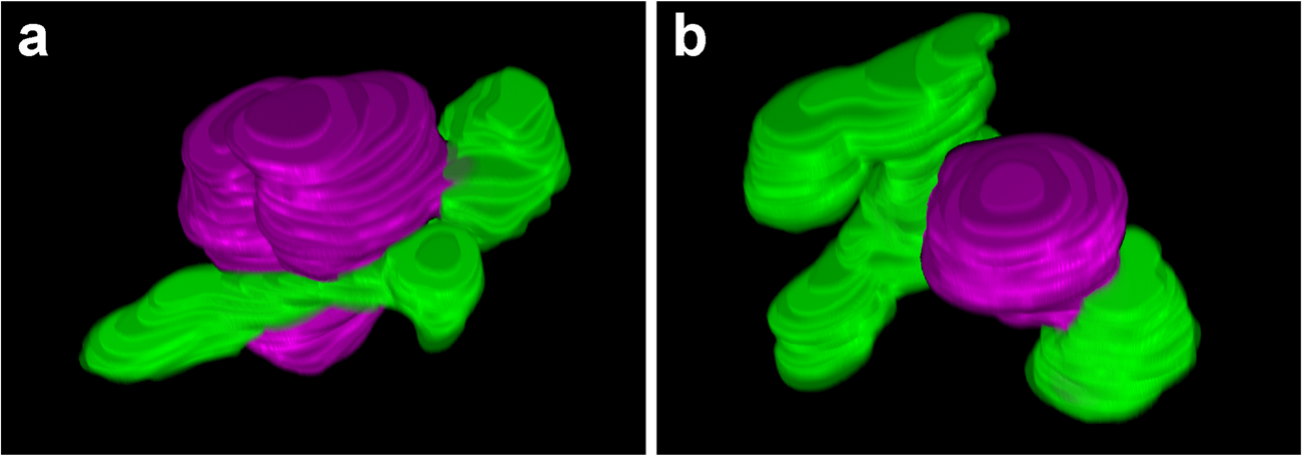
3D reconstructed images of microlipophagy. **a** and **b** were 3D images in the bicellular stage created from Fig. 4e–l and the tricellular stage created from Fig. 4m–t, respectively. Magenta structures indicate lipid bodies, and green structures indicate vacuoles

3D ultrastructural data are crucial for deepening our understanding of the interaction between lipid bodies and vacuoles. Figure 5 a and b show 3D reconstructed images created from Fig. 4e–l and Fig. 4m–t, respectively. Movie 1 and Movie 2 provide rotating animations of the 3D images in Fig. 5 a and b, respectively. Vacuoles tightly adhere to and wrap around large lipid bodies. Further, what was recognized as a separate vacuole on the section was often actually connected to a lipid body. Pollen lipophagy is a type of microautophagy in which vacuoles and lipid bodies make direct contact. Conversely, macroautophagy via an autophagosome surrounded by a double membrane also occurred in pollen (Fig. S1). The membrane structure that composes the autophagosome and the membrane structure surrounding the lipid body are clearly different in size and texture. This finding strongly supports that the structure surrounding the lipid body is a vacuole rather than endoplasmic reticulum or other similar structure.

**Fig. 6:**
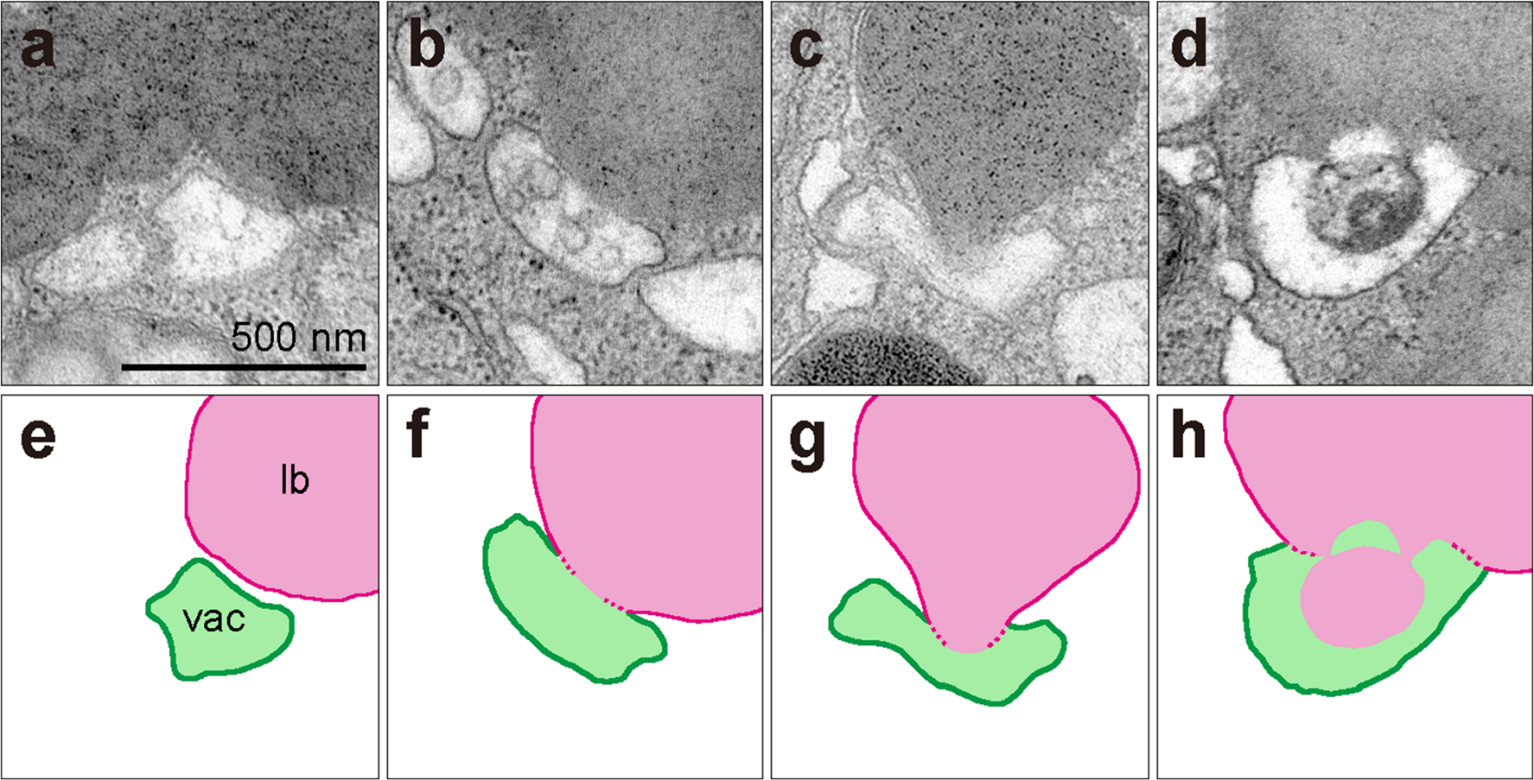
High-resolution observation of the adhesive surface between vacuoles and lipid bodies by TEM. **a**–**d** Images of vacuoles approaching, contacting, taking in, and degrading lipid bodies, arranged in the expected progression. **e**–**h** Line drawings of the photograph of **a**–**d**. Magenta lines indicate the membrane of lipid bodies (lipid monolayer), and green lines indicate tonoplast (lipid bilayer). vac, vacuoles; lb, lipid bodies. **a**–**d** have the same magnification

High-resolution observation of the contact surface between vacuole and lipid body by TEM (Fig. 6) showed that tonoplasts (shown as green lines in Fig. 6) were delineated by thick and clear lines, and lipid body membranes (shown as magenta lines in Fig. 6) displayed only a faint boundary. Tonoplasts are surrounded by a lipid bilayer, but lipid bodies are delimited by a lipid monolayer, consistent with these observations. The time series from vacuole approach to a lipid body to when the lipid body is degraded would be expected to proceed as follows. First, the vacuole closely approaches a lipid body, but a small distance (approximately tens of nanometers) remains between them (Fig. 6a). Next, the vacuole makes contact with the lipid body and rapidly fuses membranes in the contact region (Fig. 6b). The interaction region between vacuole and lipid body is only a faint boundary line, indicating that this region is not a triple layer (tonoplast plus lipid body membrane) but a monolayer or almost no layer. The vacuole then invaginates to draw in the contents of the lipid body (Fig. 6c). Subsequently, vacuoles begin to degrade lipids. The breakdown of lipids probably creates electron-dense granules, and vacuoles begin lipid degradation during lipidic content transfer, rather than first transferring the entire lipid body (Fig. 6d). Finally, most vacuoles at the tricellular pollen stage contain high electron density granules, which appear to be residues of lipid body contents (Fig. 1c).

### Abnormal microlipophagy in the *atg2-1* mutant

**Fig. 7:**
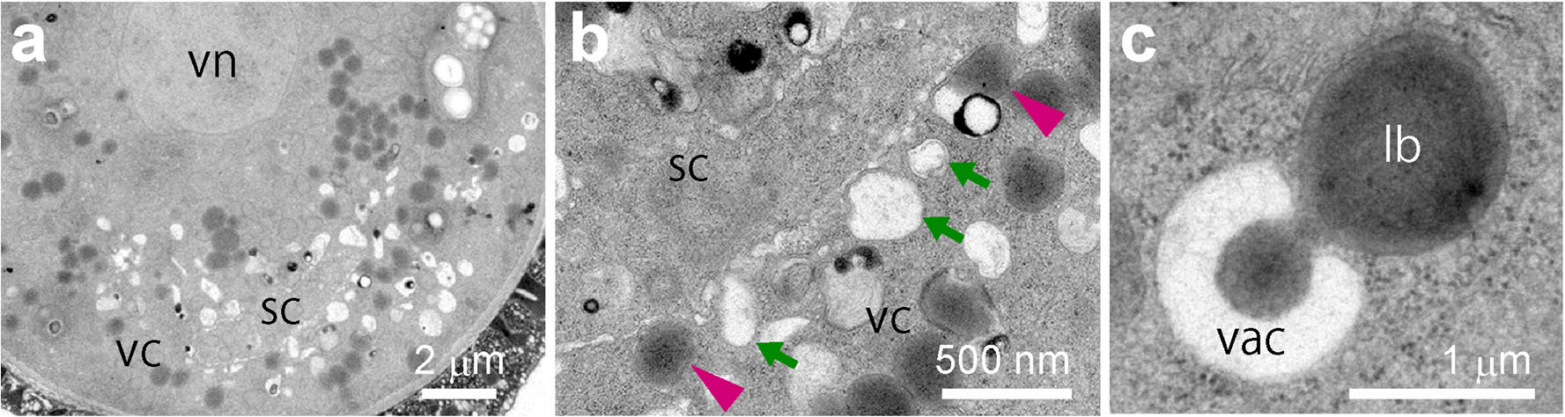
Ultrastructural analysis of *atg2-1* mutant pollen at the tricellular stage by TEM. **a**, **b** Images showing sparse vacuoles around the sperm cells. **c** Images of vacuoles drawing in lipid bodies. Magenta arrowheads indicate lipid bodies, and green arrows indicate vacuoles. vac, vacuoles; lb, lipid bodies; sc, sperm cells; vc, vegetative cells; vn, vegetative nuclei

We next used the TEM analysis of pollen from the *atg2-1* mutant to determine whether ATG2 is involved in pollen microlipophagy. The ultrastructure of the *atg2-1* mutant at the bicellular stage was similar to wild type; lipid bodies were aligned around generative cells (Fig. S2). In the early tricellular stage, the wild type had many vacuoles surrounding sperm cells; *atg2-1* mutant pollen displayed a few vacuoles (Fig. S2). High-resolution observations were made on *atg2-1* mutant pollen to clarify the interaction between lipid bodies and vacuoles (Fig. 7). The comparison of *atg2-1* mutant (Fig. 7a, b) and wild-type pollen (corresponding pollen image is shown in Fig. 1c) revealed that lipid body degradation was less complete and heterogeneous in the mutant. However, some degree of interaction between vacuoles and lipid bodies was observed in *atg2-1* pollen, similar to the interactions observed in wild-type pollen, that is, the vacuoles in *atg2-1* pollen invaginate to draw in lipid body contents (Fig. 7c). However, the contact surface between vacuoles and *atg2-1* lipid bodies is represented by a clear line, unlike structures observed in wild-type pollen. The deficiency of ATG2 probably prevents the degradation of lipid, although it does not prevent contact between organelles. Conversely, some electron-dense granules, thought to be residues of the lipid body contents, were found in vacuoles of pollen from *atg2-1* mutants (Fig. 7a, b). Mutant vacuoles are not completely incapable of lipid degradation and may degrade lipid bodies over time.

## Discussion

**Fig. 8:**
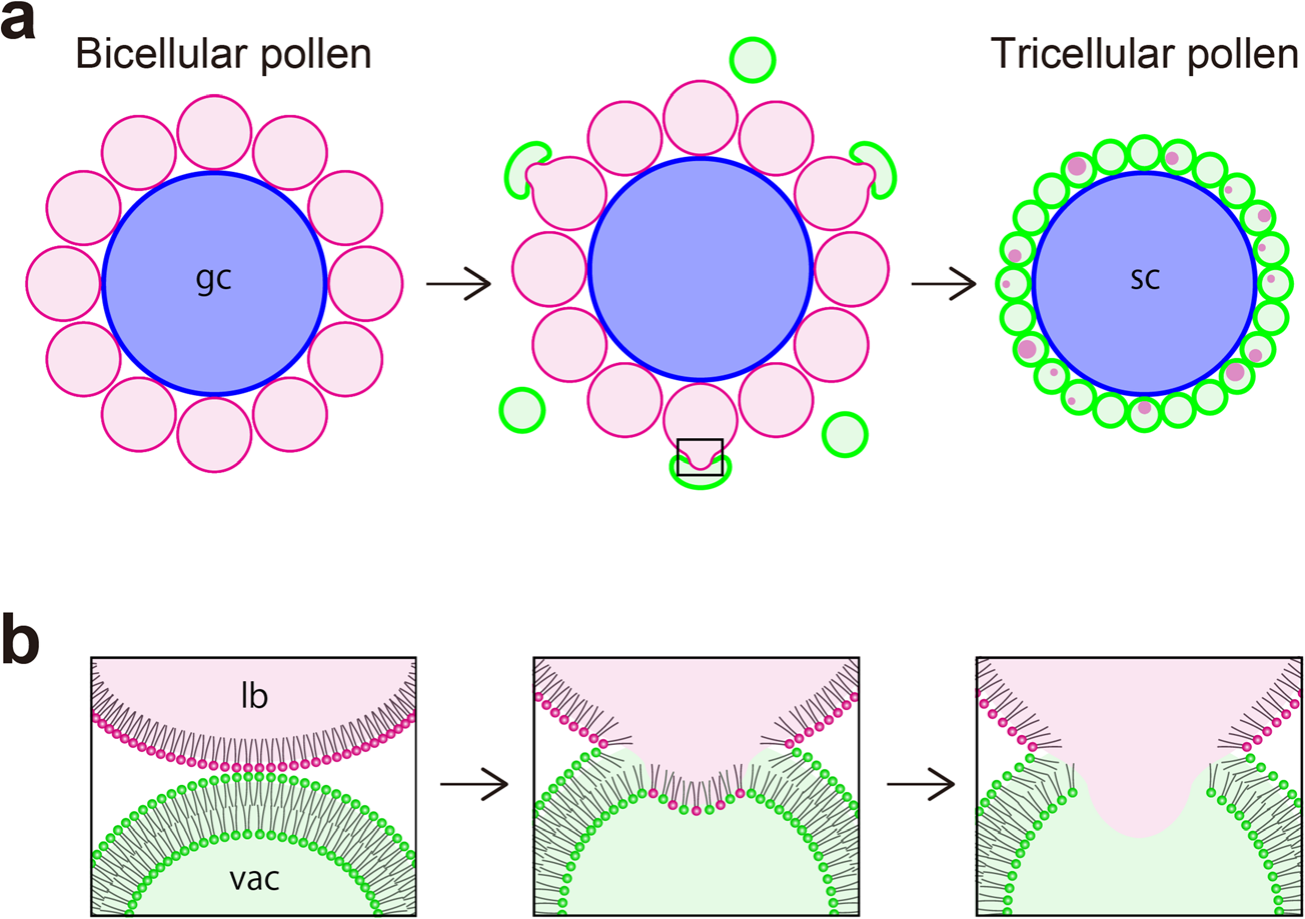
A model of microlipophagy in *Arabidopsis* pollen. **a** Characteristic distribution of vacuoles and lipid bodies in pollen. Vacuoles contact lipid bodies around generative cells, tonoplast and lipid body membranes fuse, contents of lipid bodies are drawn in and degraded, and vacuoles thus replace lipid bodies. **b** Enlarged view of the square in **a**. Expected arrangement of phospholipids of border between the vacuole and the lipid body. gc, generative cells; vac, vacuoles; lb, lipid bodies; sc, sperm cells

The characteristic distribution of vacuoles and lipid bodies in *Arabidopsis* pollen observed in our study are summarized in Fig. 8. At the bicellular pollen stage, lipid bodies in the vegetative cell line up at the surface of the generative cell. Although this phenomenon can be seen in photographs in previous reports (Owen and Makaroff 1995; Yamamoto et al. 2003), the details of this process were not described. An immediate question is why lipid bodies are closely arranged with generative cells. In the bicellular stage, generative cells are elongated and their membranes wavy (McCue et al. 2011). As generative cells expand, surrounding vegetative cell membranes must expand their surface area. Lipid bodies may supply lipid materials to cell membranes of vegetative and generative cells. Lipid bodies may also contact plasmodesmata to deliver specific enzymes to cell walls (van der Schoot et al. 2011).

Recent studies on cellular events directly involved with the distribution of lipid droplets reported cytological evidence that droplets form direct contacts with prospore membranes during meiosis II to sequester dividing nuclei in sporulating yeast (Hsu et al. 2017). Yeast cells lacking lipid droplets were severely defective in prospore membrane growth and displayed disrupted spindles, producing non-viable spores. Images of docking of lipid droplets to prospore membranes in yeast are similar to the alignment of lipid bodies around generative cells. Direct contact of lipid bodies with cell membranes in pollen may be important for proper cell membrane or wall formation between vegetative and generative cells.

We succeeded in capturing sequential changes in lipid bodies around generative cells using serial section SEM (Figs. 4 and 5). The vacuoles tightly attached to and drew in and degraded lipid bodies, replacing lipid bodies in their initial location. Shrinking lipid bodies were observed as electron-dense materials within vacuoles, likely a change in composition caused by degradation. A previous study of pollen vacuoles using cryo-fixation/freeze-substitution reported that somatic vacuoles disappeared after the second pollen mitosis and membrane-bound structures containing fine fibrillar substances (MBFs) appeared (Yamamoto et al. 2003). The relationship between somatic vacuoles and MBFs was unclear and not further explained. Our study indicates that MBFs may be vacuoles in the process of degrading lipid bodies. Rapid lipophagy occurs in tricellular pollen grains probably because it requires significant energy supply to elongate pollen tubes.

Degradation of pollen lipid bodies found in this study may be morphologically considered microlipophagy. In yeast, vacuole invagination is reported to accompany lipid droplet-specific microautophagic pathways (van Zutphen et al. 2014). During seed germination in *Arabidopsis*, microlipophagy-like processes are reported, indicating that interactions of vacuoles with lipid bodies are one mechanism for degradation of stored lipid (Poxleitner et al. 2006). Since plant cells often have large vacuoles in microautophagy, large vacuoles will contact, invaginate, and degrade small targets (Fan et al. 2019). In contrast, the relatively small vacuoles made contact with large lipid bodies in our study. In yeast, vesicles containing part of the nucleus emanate from contact sites and are degraded by vacuoles, called micronucleophagy (microautophagy of the nucleus) (Krick et al. 2009; Mijaljica et al. 2011). Microlipophagy in pollen morphologically resembles an intermediate stage of micronucleophagy. In all macroautophagy, invaginated vacuolar membranes form microdomain architecture, suggesting that microautophagy can occur by the same mechanism regardless of target size (Oku and Sakai 2018). However, in micronucleophagy, part of the target (nuclei) is picked up and degraded, but all lipid bodies are degraded in pollen microlipophagy. In pollen, lipid degradation simultaneously with transfer to vacuoles is unique and may require additional cellular machinery.

Does pollen cause only selective degradation of lipid bodies? Macroautophagy in pollen via an autophagosome surrounded by a double membrane is known (Dündar et al. 2019; Kurusu et al. 2014). Our research also revealed autophagosomes encapsulating a portion of cytoplasm (Fig. S1). Thus, non-selective macroautophagy does occur in pollen. Pollen lipid bodies might also be non-selectively degraded by macroautophagy. If we consider only microautophagy, are lipid bodies the only targets? Microlipophagy could be examined because it displays a morphologically easy-to-understand structure. In our observation of pollen, it is difficult to comprehensively understand other autophagy phenomena. Microchlorophagy (target is chloroplast) does occur in *Arabidopsis* leaves (Nakamura and Izumi 2019). We believe that various autophagy systems also occur in pollen.

The knowledge of organelle communication has increased in recent years (Dolgin 2019; Kuroiwa 2010). Interactions between organelles are ubiquitous, and membrane contact is a major route for intracellular trafficking (Valm et al. 2017). Lipid bodies interact with many cellular structures as part of cellular homeostatic mechanisms. Interactions also help buffer against starvation stress (Thiam and Dugail 2019). Yeast NPC (Niemann-Pick type C) proteins are essential for the formation and expansion of raft-like domains in vacuolar membranes. These domains engulf lipid droplets by a microautophagic mechanism (Tsuji et al. 2017). Our TEM analysis identified numbers of lipid layers by comparing membrane thickness at adhesive surfaces. Thickness at such surfaces is smaller than that of the tonoplast, indicating that this region may be a monolayer or almost no layer (Fig. 6). Chemical properties of amphoteric phospholipids may favor an arrangement of vacuoles and lipid bodies as shown in Fig. 8. This thin barrier could have a unique molecular structure similar to raft-like domains described above. This feature may facilitate the interaction, transport, and degradation of contents between organelles (Tsuji et al. 2017).

Microlipophagy in yeast shows indirect involvement of ATG genes, or macroautophagy, in microautophagic membrane dynamics (Sieńko et al. 2020). In contrast, another type of microlipophagy requires endosomal sorting complexes required for transport machinery, but not core ATG proteins (Oku and Sakai 2018). In other words, ATGs are known to be both involved and non-involved in microlipophagy. We found that the assembly of vacuoles around sperm cells of the *atg2-1* mutant was sparser, and the lipid body degradation was less complete than that in wild-type (Fig. S2; Fig. 7). Thus, ATG2 might be involved in pollen microlipophagy. However, the contribution of *ATG2* may be indirect, because the progression of microlipophagy is not entirely eliminated in mutant cells. The *atg2-1* mutant shows nearly normal fertility, and lipid bodies in pollen appear to be eventually degraded. Thus, ATG2 deficiency may cause only minor dysfunction, such as slowing the rate or reducing the frequency of degradation. More detailed analyses at a molecular level with a different approach, such as membrane labeling or quantitative analysis of lipid degradation, are required for a complete understanding of ATG involvement in microlipophagy in pollen.

High-temperature stress promoted autophagy in anther wall cells and microspores in developing anthers of WT. The *atg5-1* mutant did not show completion of tapetum degeneration and microspore maturation (Dündar et al. 2019). This and our results indicate that autophagy in *Arabidopsis* may play an important role, especially under stress. A rice mutant exhibits a more severe phenotype than *Arabidopsis*, and a *OsATG7*-knockout mutant completely abolished autophagosome-like structures and vacuole-enclosed lipid bodies (Kurusu et al. 2014). Further, vacuoles directly fused with lipid bodies were also observed in tapetal cells of rice, probably via microautophagy, but were not observed in *Osatg7* mutants (Hanamata et al. 2014). Their TEM observation that lipid bodies in *Osatg7* mutants remain in contact with vacuoles without degradation is very similar to our results in *atg2-1* mutants (Fig. 7). Also, lipidomic analyses of the rice mutant suggested impairment of editing of phosphatidylcholines and lipid desaturation during pollen maturation (Kurusu et al. 2014). Furthermore, Fan et al. (2019) also showed that microlipophagy in *Arabidopsis*, although not pollen, was suppressed in both *atg2-1* and *atg5-1*. Thus, autophagy could mediate regulation of lipid metabolism, consistent with our morphological findings that lipid body degradation appears to be inhibited in *atg2-1* mutants. Although the present study included only *atg2-1*, our study is similar to previous reports using other *atg* mutants, suggesting that the correlation between ATGs and plant microlipophagy is reliable and universal. Our study contributes to the understanding of a part of the mechanism of lipophagy and suggests a novel key feature of inter-organelle communication.

## Electronic supplementary material

Movie 1. 3D movie of microlipophagy in bicellular pollen reconstructed from images in Fig 5a. A semi-transparent image is shown. The magenta structure indicates a lipid body, and the green structure indicates a vacuole. (MP4 1924 kb)

Movie 2. 3D movie of microlipophagy in tricellular pollen reconstructed from images in Fig 5b. A semi-transparent image is shown. The magenta structure indicates a lipid body, and the green structure indicates a vacuole. (MP4 1492 kb)

Fig S1. TEM images suggesting macroautophagy in the pollen. Arrowheads indicate autophagosomes. vac, vacuoles; sc, sperm cells; vc, vegetative cells (PNG 2642 kb)

Hig Resolution (TIF 22562 kb)

Fig S2. Low magnification TEM images of pollen grains of wild type (**a, b**) and *atg2-1* mutant (**c, d**). The middle bicellular stage (**a, c**) and the early tricellular stage (**b, d**) are shown. Yellow areas indicate the generative (**a, c**) or the sperm cells (**b, d**). (PNG 1517 kb)

High Resolution (TIF 9470 kb)
