# Supplementary figures and images for: Ultrastructural characterization of microlipophagy induced by the interaction of vacuoles and lipid bodies around generative and sperm cells in Arabidopsis pollen
Source: Protoplasma. 2020 Sep 23;258(1):129–38. doi: 10.1007/s00709-020-01557-2 (PMC7782417; doi:10.1007/s00709-020-01557-2)

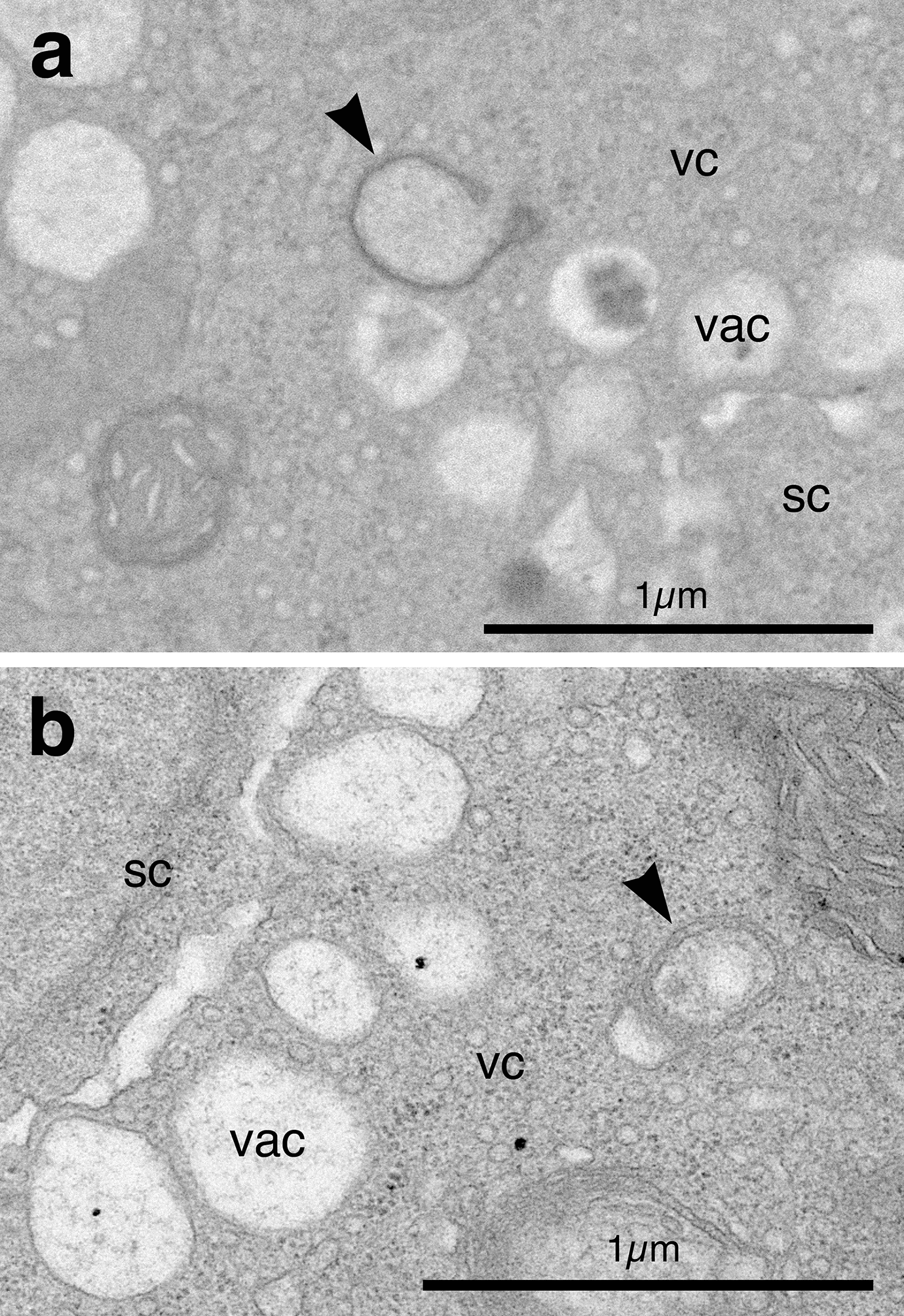

Supplement: Supplementary file 3 — TEM images suggesting macroautophagy in the pollen. Arrowheads indicate autophagosomes. vac, vacuoles; sc, sperm cells; vc, vegetative cells (PNG 2642 kb) [file 709_2020_1557_Fig9_ESM.png]

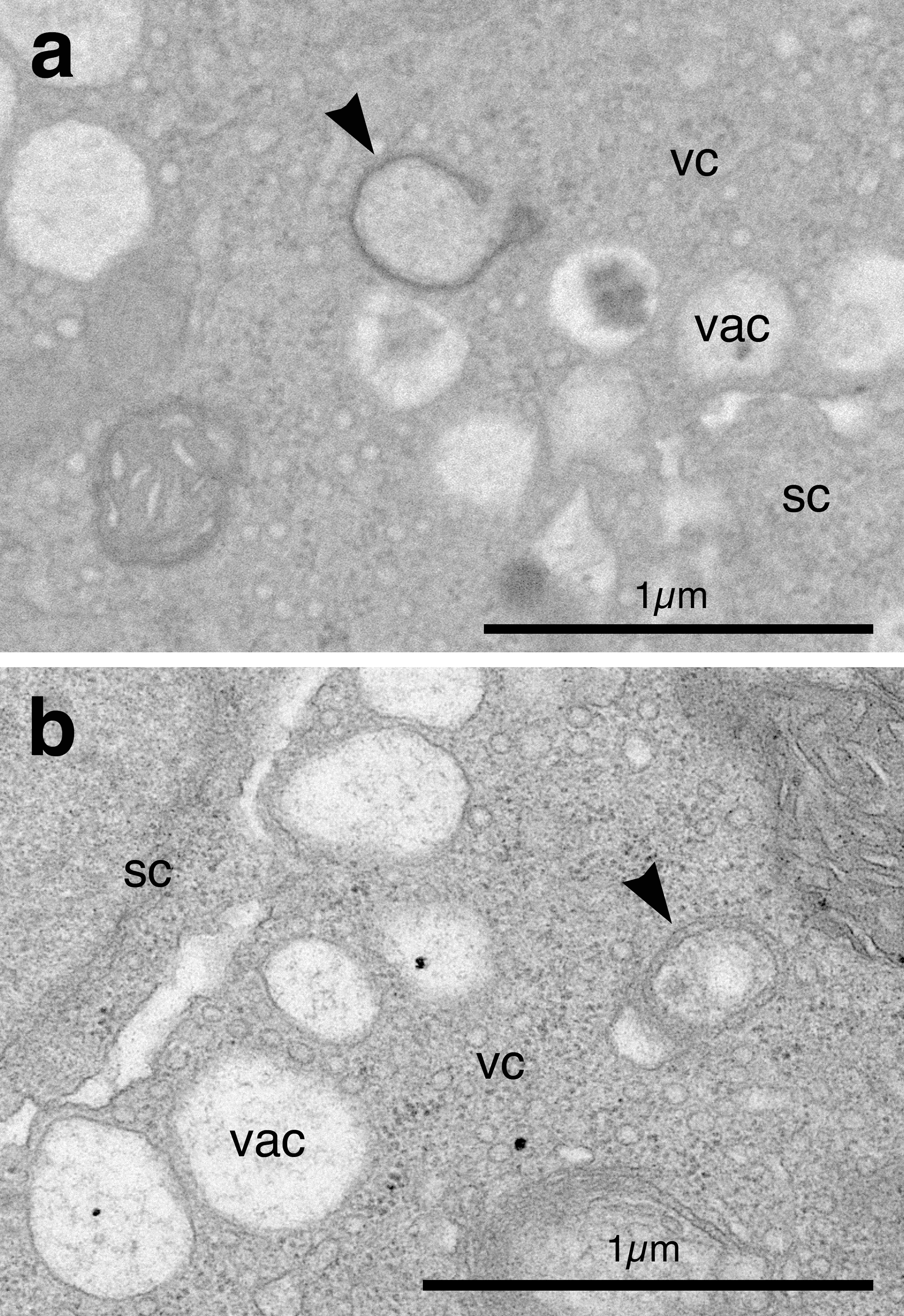

Supplement: Supplementary file 4 — Hig Resolution (TIF 22562 kb) [file 709_2020_1557_MOESM3_ESM.tif]

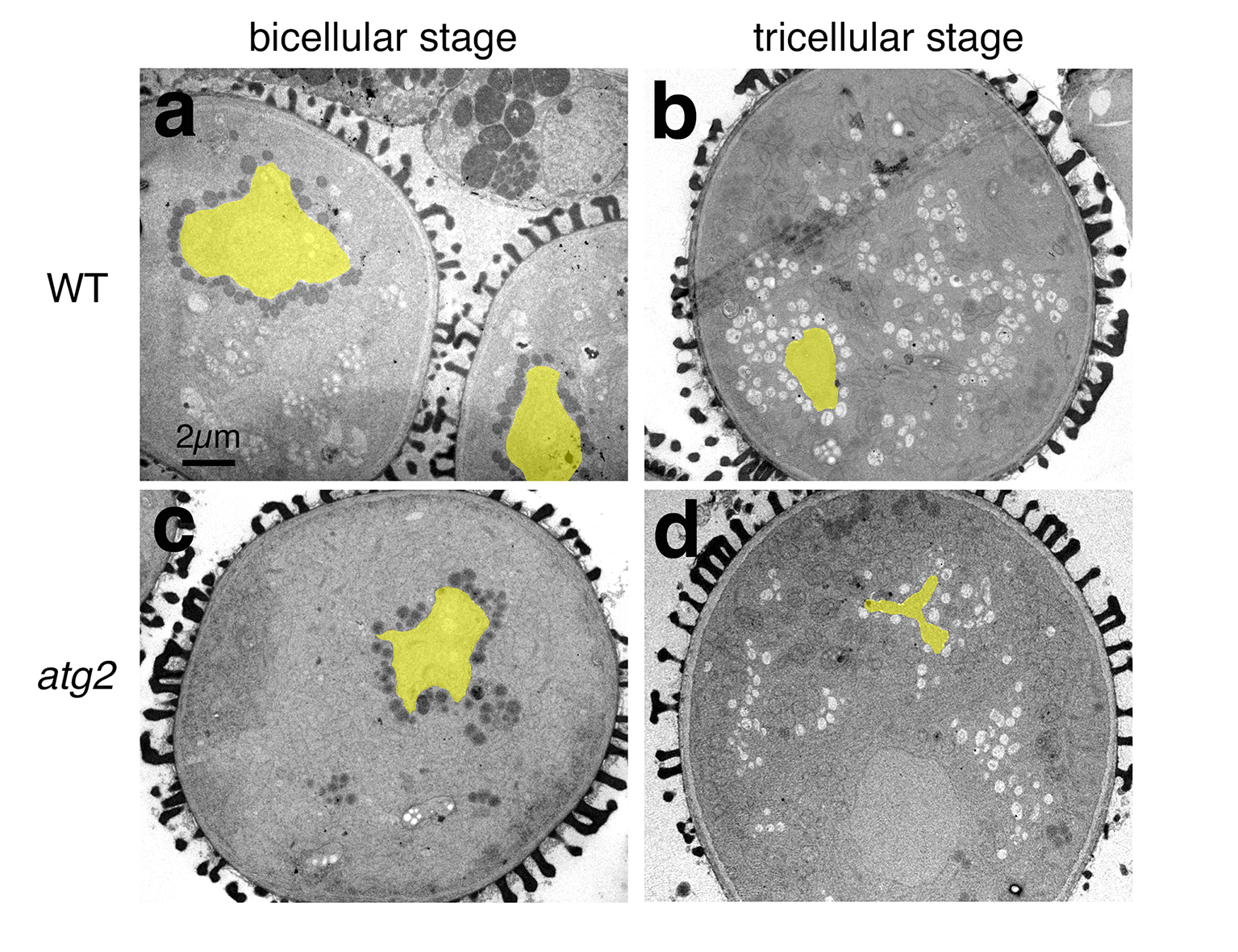

Supplement: Supplementary file 5 — Low magnification TEM images of pollen grains of wild type (a, b) and atg2-1 mutant (c, d). The middle bicellular stage (a, c) and the early tricellular stage (b, d) are shown. Yellow areas indicate the generative (a, c) or the sperm cells (b, d). (PNG 1517 kb) [file 709_2020_1557_Fig10_ESM.png]

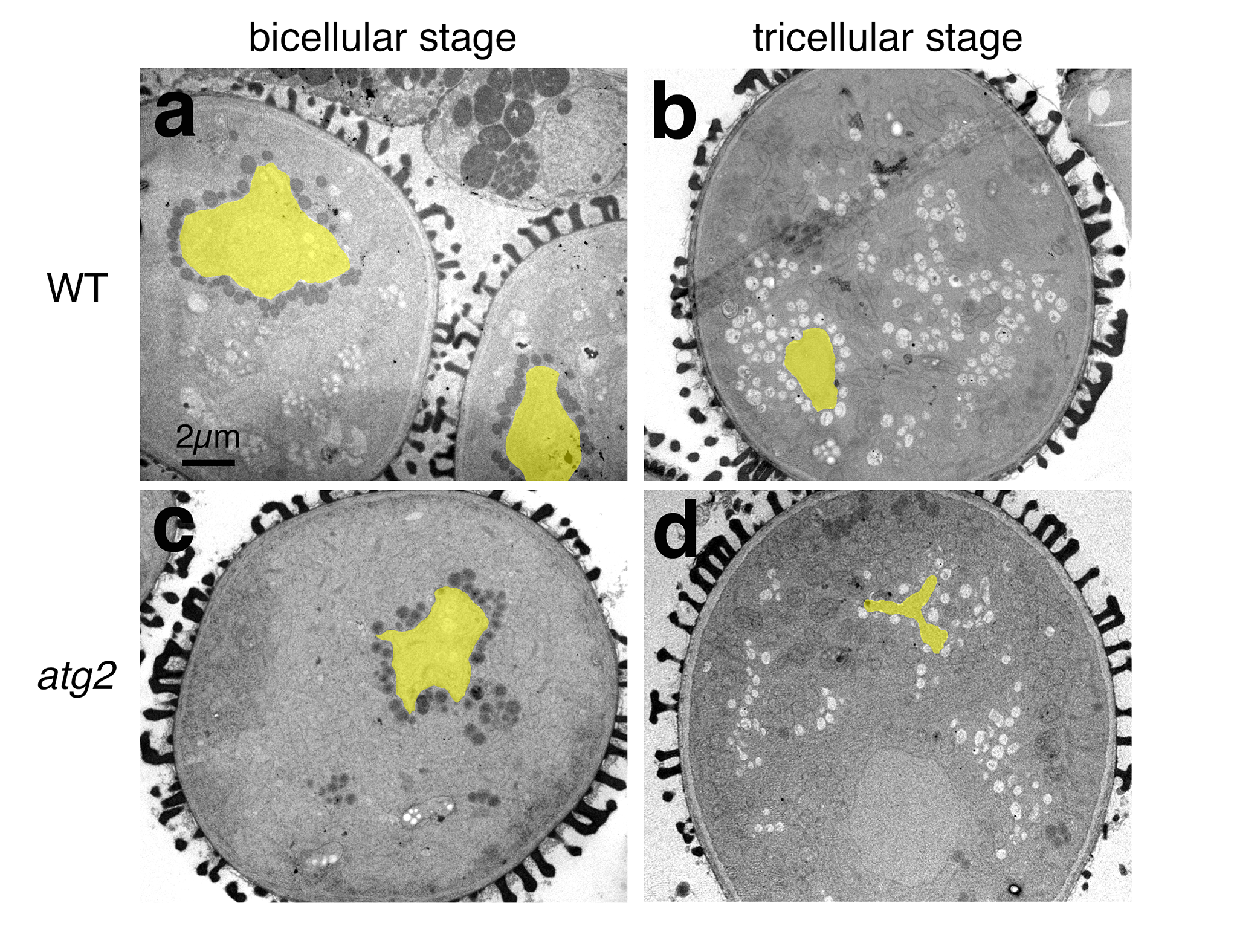

Supplement: Supplementary file 6 — High Resolution (TIF 9470 kb) [file 709_2020_1557_MOESM4_ESM.tif]
